# Effect of optimized collagenase digestion on isolated and cultured nucleus pulposus cells in degenerated intervertebral discs: Erratum

**DOI:** 10.1097/MD.0000000000013503

**Published:** 2018-11-21

In the article, “Effect of optimized collagenase digestion on isolated and cultured nucleus pulposus cells in degenerated intervertebral discs”,^[1]^ which appeared in Volume 97, Issue 44 of *Medicine*, the authors would like to note funding from the below sources:

1. The Program for Medical Key Department of Shanghai (no: ZK 2015B17);

2. The Key Disciplines Group Construction Project of Pudong Health Bureau of Shanghai (no: PWZxq2017–11).
